# Recurrent Vulvovaginal Candidiasis Caused by Fluconazole-Resistant Candida albicans: A Retrospective Study

**DOI:** 10.7759/cureus.78906

**Published:** 2025-02-12

**Authors:** Sreethish Sasi, Alaaeldin Abdelmajid, Husam Salah, Emad Ibrahim, Muna Al-Maslamani, Abdullatif Al-khal

**Affiliations:** 1 Infectious Diseases, Hamad Medical Corporation, Doha, QAT; 2 Laboratory Medicine and Pathology, Hamad Medical Corporation, Doha, QAT; 3 Biomedical Research Center, Qatar University, Doha, QAT; 4 Infectious Diseases, Hamad Medical Corporation, DOHA, QAT; 5 College of Medicine, Qatar University, Doha, QAT

**Keywords:** albicans, antifungal resistance, candida, fluconazole, vulvovaginal candidiasis

## Abstract

Background

Vulvovaginal candidiasis (VVC) is a common inflammatory condition caused by *Candida *species, primarily *Candida albicans*, manifesting with symptoms such as itching, erythema, and curd-like discharge. Recurrent vulvovaginal candidiasis (RVVC), defined by three or more symptomatic yeast infections within a year, poses significant challenges in management due to factors such as antifungal resistance and comorbidities.

Materials and methods

This retrospective descriptive study analyzed 11 cases of RVVC due to fluconazole-resistant *Candida albicans*. Data included demographic characteristics, medical history, presenting symptoms, recurrence duration, treatment modalities, comorbidities, and outcomes. Microbiological analysis involved vaginal swabs for fungal culture and antifungal susceptibility testing. The study was conducted in accordance with ethical guidelines.

Results

Patients, primarily pre-menopausal females aged 20 to 53 years, presented with vaginal itching and discharge. The mean duration of recurrent episodes, defined as the time from the initial diagnosis of RVVC to the last documented recurrence, was 1.5 years, with varied treatment responses. Comorbidities such as obesity and hypertension were noted, along with global representation.

Conclusion

RVVC is multifactorial and influenced by factors such as antifungal resistance and immune status. Diagnosis relies on comprehensive evaluation and microbiological testing. Effective management of RVVC requires personalized strategies and long-term monitoring. Despite challenges, individualized approaches can optimize outcomes and enhance the quality of life of affected individuals. Further research is warranted to refine diagnostic and therapeutic strategies.

## Introduction

Vulvovaginal candidiasis (VVC) is characterized by inflammation within the vaginal and vulvar regions, caused by *Candida *species, primarily *Candida albicans* [1]. Symptoms include itching, erythema, and curd-like discharge [1]. Unlike oropharyngeal candidiasis, VVC is not considered an opportunistic infection and is not categorized as a sexually transmitted disease [2]. Diagnosing VVC can be intricate, as symptoms may overlap with other gynecological conditions [3]. The gold standard for diagnosis is microscopic examination [3]. Recent advances in molecular and point-of-care tests offer quicker results [3]. Self-diagnosis of VVC is discouraged as it is inaccurate and leads to inappropriate, over-the-counter treatments [4]. Recurrent vulvovaginal candidiasis (RVVC) is characterized by three or more symptomatic yeast infections within a year [5]. RVVC affects 138 million women annually, with a global annual prevalence of 3,871 per 100,000 women, with the highest being in women aged 25 to 34 years [5]. Asymptomatic patients with *Candida* vaginal colonization do not need treatment [2,5]. Exact causes of RVVC are unclear, but factors such as microbiology, altered immune responses, genetic polymorphisms, bacterial vaginosis, mannose-binding lectin (MBL) and interleukin-4 (IL-4) deficiencies, antibiotics, sexual activity, and diet contribute to it [5,6]. This study aims to analyze the clinical and microbiological characteristics of RVVC caused by fluconazole-resistant *Candida albicans*. Additionally, it seeks to evaluate treatment outcomes and highlight challenges in managing RVVC in clinical practice, with a focus on antifungal resistance and the role of individualized treatment approaches.

## Materials and methods

This retrospective descriptive study was conducted at a sexual health clinic affiliated with Hamad Medical Corporation (HMC) in Doha, Qatar, between August 2022 and March 2024, to analyze the clinical and microbiological characteristics of RVVC caused by fluconazole-resistant *Candida albicans*. The study included 11 pre-menopausal women aged 18 years or older who experienced three or more symptomatic yeast infections within a year, confirmed by fungal culture and antifungal susceptibility testing. No patients were excluded. Data extracted from electronic medical records included demographic details, medical history, presenting symptoms, recurrence duration, treatment modalities, microbiological findings, and clinical outcomes, with follow-up assessments at 1, 3, 6, and 12 months. RVVC was diagnosed based on clinical presentation and microbiological confirmation. A standardized clinical form was used to record patient demographics, comorbidities, recurrence history, treatment regimen, and follow-up findings.

Vaginal swab samples were collected using sterile techniques, processed for fungal culture on Sabouraud dextrose agar (SDA) and CHROMagar Candida plates, and incubated at 37°C for 48 to 72 hours, with species identification confirmed by matrix-assisted laser desorption ionization time-of-flight mass spectrometry (MALDI-TOF MS). Antifungal susceptibility testing was performed using broth microdilution as per CLSI M27-A3 and M60 guidelines. The following antifungal agents and their breakpoints were tested: fluconazole (≥8 µg/mL for resistance), amphotericin B (≥2 µg/mL), anidulafungin, micafungin, and caspofungin. Quality control was ensured using *Candida parapsilosis* (Ashford) Langeron et Talice - 22019 (ATCC® 22019™) and *Candida krusei* (ATCC™ 6258™) reference strains. No significant anomalies were observed during testing.

Patients were treated according to institutional and international RVVC guidelines. High-dose induction therapy included amphotericin B (3-5 mg/kg IV daily), anidulafungin (200 mg IV loading, followed by 100 mg daily), or caspofungin (70 mg IV loading, then 50 mg daily) for 7-14 days. Maintenance therapy included weekly oral fluconazole (150-200 mg) for six months or topical boric acid (600 mg vaginal suppository daily for 14 days and then twice weekly for six months). Switching was based on clinical response, with patients exhibiting persistent symptoms on azoles shifted to non-azole regimens. Symptom improvement was assessed using a standardized scale (visual analog scale, 0-10 for itching and discomfort), and treatment success was defined as negative cultures at 6 and 12 months with complete symptom resolution. Adjunctive measures included counseling on lifestyle modifications such as dietary changes, avoidance of irritants, and proper hygiene practices. Follow-up evaluations monitored treatment response, categorizing outcomes as complete cure (symptom resolution and negative cultures), partial response (symptom improvement with persistent positive cultures), relapse (symptom recurrence with positive cultures), or lost to follow-up. Ethical approval was granted by the Institutional Review Board at HMC (approval number: MRC-01-24-159), with informed consent waived due to the retrospective nature of the study and the use of anonymized data.

Statistical analysis

Descriptive statistics were used to summarize demographic, clinical, and microbiological data. Continuous variables, such as age and recurrence duration, were expressed as medians with interquartile ranges (IQRs), while categorical variables, including comorbidities and treatment outcomes, were presented as frequencies and percentages. Due to the small sample size, inferential statistical analyses were not performed.

## Results

Eleven cases of RVVC due to fluconazole resistant *Candida albicans* were analyzed. Variations in patient characteristics and outcomes were observed despite a common pathogen. Clinical features of the 11 cases are consolidated in Table 1.

**Table 1:** Summary of clinical and microbiological characteristics of the 11 cases

Case No.	First presentation	Age	Nationality	Comorbidities	HbA1c	Relationship status	Symptoms	Duration of symptoms and number of recurrent episodes	Previous fluconazole treatment	Previous fluconazole chronic suppressive therapy	Diagnostic modality	Sensitivity pattern of Candida albicans	Allergies	Treatment given	Outcome
1	August 2022	41	Tunisia	None	5.5%	Monogamous married relationship for last 11 years	Vaginal itching, whitish vaginal discharge	1 year, 6 episodes	Yes	Yes	Genital Culture (high vaginal swab)	Amphotericin B - S Anidulafungin - S Micafungin - S Caspofungin - S Fluconazole - R Itraconazole - R Ketoconazole – R Posaconazole - R	Anidulafungin	Amphotericin B IV for 5 days followed by boric acid vaginal suppositories for 10 days	Cured but relapsed after 6 months, but this time fluconazole-sensitive C. albicans
2	August 2022	53	Qatar	Obesity, hypertension	5.6%	Monogamous married relationship	Vaginal itching, whitish vaginal discharge, uterine polyp	2 years, 8 episodes	Yes	No			None	Anidulafungin for 7 days	Cured with no relapse
3	January 2023	33	Netherlands	None	5.1%	Single sexual partner for last 10 months	Redness and itching of vagina, with whitish curdy discharge	8 months, 4 episodes	Yes	Yes			None	Anidulafungin for 7 days followed by boric acid vaginal suppositories for 14 days	Cured but relapsed after 3 months. Using boric acid suppositories with new episodes
3	May 2023	44	Qatar	Obesity post Gastric sleeve and bypass	5.4%	Monogamous married relationship	Vaginal itching, curdy vaginal discharge	1.5 years, 6 episodes	Yes, vaginal clotrimazole pessaries/itraconazole and fluconazole	No			None	Caspofungin for 14 days	Cured, but relapsed after 5 months
4	October 2023	35	Kenya	None	5.5%	Monogamous married relationship	Vaginal itching, curdy vaginal discharge	1.5 years, 4 episodes	Yes, monthly fluconazole for last 6 months	Yes			None	Topical nystatin cream for 14 days followed by boric acid vaginal suppositories for 14 days	Cured
6	October 2023	28	Qatar	None	5.5%	Monogamous married relationship for 10 years	Vaginal itching, curdy vaginal discharge	1 year, 4 episodes	Yes, 6 courses of 1 week each in the last year	No			None	Anidulafungin for 7 days followed by boric acid vaginal suppositories for 14 days	The patient's symptoms resolved temporarily, but she returned after one month with symptoms of cervicitis. A urine PCR test at this time confirmed the presence of *Chlamydia trachomatis*
7	November 2023	20	Qatar	Had co-infection with *Mycoplasma hominis*	NA	Single male sexual partner for last 1 year	Cheesy-like greenish thick vaginal discharge	1 year, 5 episodes	No	No			None	For *Mycoplasma hominis*, oral doxycycline for 1 week followed by oral moxifloxacin for 1 week. For *Candida albicans*, intravenous anidulafungin for 2 weeks	Patient did not turn up for follow-up for results of C. albicans resistance testing
8	December 2023	37	Qatar	History of peri-partum cardiomyopathy	5.4%	Monogamous married relationship	Vaginal itching	6 months, 3 episodes	Yes	No			None	Anidulafungin for 7 days	No follow-up
9	December 2023	38	Qatar	Had co-infection with *Mycoplasma hominis*	5.1%	Monogamous married relationship	Vaginal itching, whitish vaginal discharge	2 years, 9 episodes	Yes	Yes			None	IV anidulafungin for 2 weeks followed by boric acid vaginal suppositories for 2 weeks	No improvement with anidulafungin but improved with boric acid. Relapsed after 3 months but again cured by boric acid suppositories.
10	February 2024	31	Qatar	Obesity post-gastric sleeve and bypass	5.2%	Monogamous married relationship	Cheesy-like greenish thick vaginal discharge, vaginal itching, and burning sensation	1 year, 5 episodes	Yes	No			Anidulafungin	Boric acid suppositories for 2 weeks	No follow-up
11	March 2024	28	Kenya	None	5.2	Single male sexual partner in the previous year (uses condoms)	Whitish or greenish vaginal discharge with vaginal itching	1 year, 4 episodes	Yes	No			None	Anidulafungin for 7 days followed by boric acid vaginal suppositories for 14 days	Cured

The study population consisted of pre-menopausal females with ages ranging from 20 to 53 years. The median age was 35 years, with an interquartile range of 28 to 38 years. Comorbidities were reported in some cases, including obesity, hypertension, and a history of gastric sleeve and bypass surgery. Two cases involved co-infection with *Mycoplasma hominis*. The patients hailed from the Middle East (Qatar, Tunisia), Africa (Kenya), and Europe (the Netherlands), indicating a global prevalence of RVVC. In terms of clinical presentation, vaginal itching and discharge emerged as ubiquitous symptoms across all cases, underscoring their significance as hallmark features of RVVC. The recurrence duration, averaging around 1.5 years, points to the chronic nature of the condition. Previous fluconazole treatment was reported in all cases. Some patients had also received chronic suppressive therapy with fluconazole. Treatment modalities vary, encompassing the use of diverse antifungal agents such as amphotericin B, anidulafungin, micafungin, and caspofungin, highlighting the necessity for tailored therapeutic approaches. The absence of multiple sexual partners or high-risk sexual behaviors in any of the 11 cases suggests that it does not contribute as an additional risk factor in the development of RVVC. Treatment outcomes varied, with cases 1, 3, 4, 6, and 9 experiencing initial cures but subsequent relapses, suggesting the need for long-term monitoring. In particular, case 1 relapsed with fluconazole-sensitive *Candida albicans*, and case 9 initially showed no improvement with anidulafungin but responded to boric acid. In contrast, cases 2, 5, and 11 achieved complete cures without relapse, indicating favorable responses to the treatment. Cases 7, 8, and 10 did not attend follow-up, complicating the assessment of long-term outcomes.

## Discussion

Uncomplicated VVC is defined by features such as less than three episodes per year, non-pregnant status, mild-to-moderate symptoms, probable *Candida albicans* infection, and immunocompetent individuals [1]. Oral and topical fluconazole provide symptom relief within 24 hours and infection resolution in 80-90% of patients [1- 4]. RVVC is widely recognized as a multifactorial disorder influenced by factors such as species of *Candida*, lactobacilli population, microenvironment, genetic factors, immune status, behavioral factors, pregnancy, contraceptives, diabetes mellitus, antibiotic use, and sexual intercourse [5]. While our findings suggest that factors such as obesity and prior antibiotic use may contribute to recurrence, our small sample size does not allow us to draw definitive conclusions regarding their overall impact. Larger, controlled studies are needed to clarify these relationships. Fungal infections of the lower genital tract decrease during menopause, except for women using hormonal replacement therapy and post-menopausal women with poorly controlled diabetes. Vaginal estrogen levels also play a role in predisposing women to the disease [1,3]. All patients in this study were pre-menopausal, with ages ranging from 20 to 53 years, and none of them were diabetic. Predisposing factors are absent in 20%-30% of patients with RVVC [3]. Moreover, 63% of the patients in this series had no predisposing factors. Co-infection with *Mycoplasma hominis* was noted in cases 7 and 9, highlighting the potential role of concurrent infections in RVVC. Although our study included patients from the Middle East, Africa, and Europe, the majority were from Qatar, and the sample size remains small. While our findings may reflect regional trends, they should not be extrapolated to global populations without further research in diverse clinical settings.

*Candida albicans* is a diploid, polymorphic fungus with yeast, pseudohyphae, and filament forms, while *Candida glabrata* is haploid and grows only in yeast form [6]. Over time, these species can develop antifungal drug resistance through mechanisms such as overexpression of membrane transporters, altered ergosterol biosynthesis, altered sterol and azole import, genomic variations, and chromosomal alterations [6]. These mechanisms decrease their susceptibility to therapies and increase the risk of recurrent episodes of vaginal candidosis [6].

The 2018 Sexually Transmitted Infections (IUSTI) - World Health Organization (WHO) guideline on managing vaginal discharge recommends microscopy as the best test for diagnosing VVC [7]. Full susceptibility testing should also be included [7]. To confirm the diagnosis of RVVC, at least one symptomatic episode should be confirmed by culture and species identification [7,8]. Diagnosis of VVC involves clinical features, microscopic detection of pseudo-hyphae and blastopores, and cultures in unclear cases [8]. Patient history is crucial for understanding previous positive tests, treatment response, and risk factors for recurrence [7,8]. In our study, all cases were diagnosed by vaginal swab or discharge culture and sensitivity.

Clinical practice guidelines for RVVC recommend topical and oral azoles for induction therapy and oral fluconazole as the first option for maintenance therapy [7-11]. The diagnosis and treatment with fluconazole occur simultaneously to reduce patient burden and healthcare system costs [8]. The 2018 European IUSTI/WHO guideline [7] recommends an initial intensive regime of 150-200 mg daily for three days to attempt mycologic remission before initiating a maintenance regimen for six months. The Association of the Scientific Medical Societies in Germany recommends a dose-reducing suppression therapy for RVVC, based on the ReCiDiF regimen (individualized decreasing-dose maintenance fluconazole regimen for RVVC) [9]. This treatment allows patients to move to the next level of maintenance only if they are symptom-free and have negative microscopy and culture results [9]. The ReCiDiF regimen has shown that up to 79% of women are free of disease at 12 months [9]. RVVC treatment requires accurate diagnosis, treatment recommendations, patient education, and adherence to therapy [7-10]. Azole therapy is the most common first-line treatment, but it is insufficient for resistant *Candida* strains. Treatment resistance with *Candida albicans* is rare, dose-dependent, and difficult to test [7-10]. Brazilian [10] and European [7] guidelines recommend topical nystatin for vulvovaginitis caused by fluconazole-resistant and non-albicans *Candida *species. Brazilian guidelines suggest topical boric acid for *Candida glabrata *RVVC, while European guidelines recommend nystatin as a first-line therapy [10]. Treatment with first- or second-generation azoles for *Candida glabrata* infection is usually unsuccessful, and thus local nystatin, ciclopirox olamine, or boric acid is recommended [7,10]. However, boric acid may cause fertility impairment and embryotoxicity during pregnancy [7]. Topical nystatin is effective for chronic RVVC caused by non-albicans and fluconazole-resistant species, with a mycological cure rate reported in 64.3% and 55% of patients [7-10].

The increasing number of azole-resistant cases necessitates new, affordable, and effective drugs. Oteseconazole, an oral selective inhibitor of fungal lanosterol demethylase, has shown strong activity against azole-resistant *Candida glabrata*, but its use is limited to postmenopausal patients, those who have undergone bilateral tubal ligation, or those who have undergone hysterectomy [11]. It should not be used in patients of reproductive potential, including those who could become pregnant, are pregnant, or are lactating [11]. Other novel antifungal agents, such as ibrexafungerp, topical miconazole, and an immunotherapeutic vaccine (NDV-3A), are also being developed [12]. In cases of dose-dependent antifungal resistance, ReCiDiF could be beneficial [9]. Ibrexafungerp, an oral triterpenoid drug, is another option for suppressing RVVC for patients who have not responded to azoles, have not tolerated azoles, or have an azole-resistant infection [12]. It has shown activity against azole-resistant and echinocandin-resistant *Candida* strains. It is highly bioavailable and has shown sustained symptom resolution in patients with acute VVC [12]. The FDA-approved ibrexafungerp for post-menarcheal females with VVC in June 2021, with a recommended dosage of 300 mg [12]. A suppressive regimen combined with intra-uterine device removal resolved vulvovaginitis symptoms [12].

Impact of *Candida* VVC and asymptomatic vaginal colonization on pregnancy outcomes is controversial. Available studies on this topic are summarized in Table 2 [13-19].

**Table 2:** Literature review on the impact of vaginal candidiasis on pregnancy outcomes BV, bacterial vaginosis; PVD, pathological vaginal discharge; VVC, vulvovaginal candidiasis

Study	Participants	Main findings	Implications	Negative impact on pregnancy outcome
Holzer et al. [13]	1,066 pregnant women	Preterm birth rate higher in women colonized with *Candida* in the second trimester (18%) compared to the first trimester (10%). Mean neonatal birth weight lower in the second trimester colonization group.	Screening programs for asymptomatic *Candida* colonization should consider the timing of colonization during pregnancy.	Yes
Farr et al. [14]	8447 pregnant women	Recurrent asymptomatic *Candida* colonization in early pregnancy associated with higher rates of preterm delivery and low birth weight.	Highlights the importance of routine screening and treatment for candidiasis in improving pregnancy outcomes.	Yes
Roberts et al. [15]	685 pregnant women (from RCTs)	Treatment of asymptomatic vaginal candidiasis associated with significant reduction in spontaneous preterm births. Results from post-hoc subgroup analysis.	Suggests the potential benefit of treating asymptomatic candidiasis in preventing preterm birth, but further prospective trials are needed for confirmation.	Yes
Sule-Odu et al. [16]	408 pregnant women	Higher prevalence of *Candida* infection in early labor (46%). *Candida albicans* predominant. Associated with increased risk of low-birth-weight babies. No significant effect on preterm delivery.	Recommends routine screening and prompt treatment for women at risk of delivering low birth weight babies.	Yes
Khaskheli et al. [17]	85 pregnant women	Most participants had vaginal discharge (89%), with 69.7% having infections. PVD associated with adverse maternal and perinatal outcomes.	Emphasizes the frequency of PVD and its association with adverse outcomes, highlighting the need for appropriate management.	Yes
Hizkiyahu et al. [18]	204 pregnant women	102 women with vaginal *Candida* during pregnancy (of whom 70% had *Candida albicans *species) and 102 controls with normal vaginal flora. No significant differences in pregnancy and delivery outcomes between *Candida-*positive and negative women. No increased risk of obstetrical tears in the *Candida-*positive group.	*Candida* colonization during pregnancy not associated with increased risk of obstetrical tears.	No
Hu et al. [19]	793 pregnant women	Different trends of VVC, trichomoniasis, and BV during pregnancy not associated with adverse outcomes except for persistent BV.	Persistent BV throughout pregnancy associated with adverse outcomes, indicating the need for monitoring and management.	No

While some studies suggest associations with adverse outcomes such as low birth weight and preterm delivery [13-17], others indicate no significant effects [18,19]. Routine screening and appropriate management of vaginal candidiasis, especially in early pregnancy, could potentially improve pregnancy outcomes, but further research is warranted to establish causality and modify clinical practice. During pregnancy, treatment is considered complicated due to potential fetal risks associated with some drugs [13-19]. For pregnant patients, topical antifungals (clotrimazole, miconazole, or nystatin suppositories) are recommended for seven days due to the potential risks associated with oral antifungal agents [13-19]. The benefits and disadvantages of simultaneous contraception should be discussed with patients [17,19]. Treatment duration in clinical practice guidelines is three to six months, with 50% to 70% of women relapsing after stopping maintenance [17,19]. Maintenance therapy can continue beyond six months, even years in some problematic cases [13-19].

The presented cases of RVVC provide valuable insights that align with existing guidelines for managing the condition. The multifactorial nature of RVVC, including factors such as antifungal resistance, comorbidities, and immune status, is illustrated in real-world scenarios. Treatment responses varied despite the prevalence of fluconazole-resistant *Candida albicans* strains, underscoring the need for individualized therapeutic approaches. The diversity in patient demographics, including nationality and comorbidities, mirrors the guidelines' emphasis on tailoring treatment strategies to each case. The challenges in diagnosis also align with the guidelines' recommendations for comprehensive evaluation, including microbiological testing and risk factors.

Limitations

This study has several limitations that impact its generalizability and clinical applicability. The retrospective design inherently limits our ability to control for confounders and assess causal relationships. Additionally, reliance on electronic medical records introduces potential biases due to variability in documentation quality and completeness. The small sample size (n=11) restricts our ability to draw robust statistical conclusions and detect meaningful patterns in treatment response or resistance trends. The lack of a control group prevents direct comparisons between fluconazole-resistant and fluconazole-susceptible RVVC cases. Moreover, being a single-center study in Qatar, the findings may not be generalizable to broader populations, particularly in different geographic and socioeconomic settings. Another limitation is the loss to follow-up in three cases, which hampers long-term outcome assessments. The retrospective nature also limits data on treatment adherence, potential side effects of antifungal regimens, and patient-reported symptom burden, all of which could influence treatment success rates. Future studies should adopt a prospective, multicenter approach, incorporating larger sample sizes, standardized clinical assessment tools, and longitudinal follow-ups to provide a more comprehensive understanding of RVVC management. Additionally, exploring host immune responses and genomic variations in resistant *Candida *strains could offer deeper insights into resistance mechanisms.

Strengths

Despite these limitations, our study has several key strengths. The comprehensive microbiological analysis, including species identification via MALDI-TOF MS and broth microdilution antifungal susceptibility testing, ensured precise characterization of fluconazole-resistant *Candida albicans* strains. This contributes to the growing body of literature on emerging antifungal resistance trends. The study also provides real-world clinical insights into treatment responses and resistance patterns, with detailed documentation of individualized antifungal regimens. The extended follow-up period (up to 12 months) allowed us to capture relapse rates and long-term treatment efficacy, reinforcing the importance of maintenance therapy. Additionally, the diverse patient demographics (Middle East, Africa, Europe) improve the study's global relevance, highlighting RVVC as a widespread condition beyond specific geographic boundaries. Our findings emphasize the need for tailored antifungal regimens, particularly in fluconazole-resistant cases, where alternatives such as boric acid and echinocandins demonstrated efficacy. By incorporating these findings into clinical practice, we hope to aid in the development of standardized treatment guidelines for fluconazole-resistant RVVC. Future research should focus on novel antifungal agents, host-pathogen interactions, and personalized treatment strategies to optimize patient outcomes.

## Conclusions

RVVC is a multifactorial condition influenced by immune status, microbiological factors, and comorbidities, as noted in existing literature. Our study suggests that obesity and prior antibiotic use may contribute to recurrence, but the small sample size limits definitive conclusions. Larger, controlled studies are needed to clarify these relationships. The retrospective design also restricts our ability to assess long-term treatment effectiveness and adherence, compounded by some patients being lost to follow-up, raising uncertainty about sustained responses. Future prospective studies with extended follow-up periods are essential to validate treatment durability. Recurrent *Candida albicans *vulvovaginitis requires personalized strategies, considering comorbidities and treatment history. While antifungal sensitivity patterns guide therapy, long-term success is not guaranteed, and chronic fluconazole suppressive therapy is not universally effective. Continuous patient monitoring is crucial, as adjustments may be needed for sustained relief. This study underscores the importance of a patient-centered approach to optimize outcomes and improve quality of life. Our findings offer insights into managing fluconazole-resistant RVVC, highlighting the need for individualized strategies and the limitations of fluconazole monotherapy. However, the small sample size and retrospective design prevent definitive conclusions on long-term outcomes. Although the inclusion of patients from multiple regions suggests broader relevance, these findings require cautious interpretation and validation through larger, prospective studies. Future research should focus on optimizing antifungal regimens, exploring host-pathogen interactions, and identifying predictive markers for treatment response.
